# The Many Roles of the Rock: A Qualitative Inquiry into the Roles and Responsibilities of Fathers of Children with Brain Tumors

**DOI:** 10.3390/children6100113

**Published:** 2019-10-11

**Authors:** Jacob E. Robinson, David Huskey, Jonathan Schwartz, Meaghann S. Weaver

**Affiliations:** 1 Division of Pediatric Palliative Care, Department of Pediatrics, Children’s Hospital and Medical Center, Omaha, NE 68114, USA; jacober@live.unc.edu; 2 Division of Spiritual Care and Ministry, Department of Pediatrics, Children’s Hospital and Medical Center, Omaha, NE 68114, USA; dhuskey@childrensomaha.org; 3 Division of Neuro-Oncology, Department of Pediatrics, Children’s Hospital and Medical Center, Omaha, NE 68114, USA; jonathan.schwartz@unmc.edu

**Keywords:** fathers, pediatric neuro-oncology, pediatric palliative, caregivers, qualitative research

## Abstract

A pediatric brain tumor diagnosis impacts an entire family unit, from diagnosis through curative treatment, and into survivorship or bereavement. Paternal caregiver experience has been significantly underexplored in pediatric neuro-oncology research as compared to maternal experience. This case series study explores the paternal roles, responsibilities, strengths, challenges, personal growth, and support needs of fathers of children with brain tumors receiving new palliative care consultations. In the study setting, a neuro-oncology diagnosis results in an automatic referral to the palliative care team, and thus, a convenience sampling model was employed based on consecutive palliative care consults for new childhood brain tumor diagnoses. In this study, four fathers of pediatric brain tumor patients receiving palliative care consultations responded to eight open-ended questions. Individual, voice-recorded interviews were transcribed for semantic content qualitative analysis. Analysis followed Consolidated Criteria for Reporting Qualitative Research (COREQ) guidelines. Participants completed quantitative surveys of their information preferences and support needs. Participants defined their father role as: being a team parent, an adaptable father, supporter, provider, a present father, and protector. Role conflict due to paternal responsibilities were recognized, such as the absence from the hospital to provide financial security for the family, and yet a desire to be physically present for the child. Fathers prioritized their knowledge needs about their child’s diagnosis, prognosis, and treatment above emotional needs. Fathers shared experiences of their personal growth through their child’s brain tumor diagnosis and advised on preferred support formats to include both verbal and written information. Understanding how paternal caregivers of children with cancer define their roles and goals has potential to improve the care and communication delivered to families of pediatric neuro-oncology patients.

## 1. Introduction

**Figure 1:**
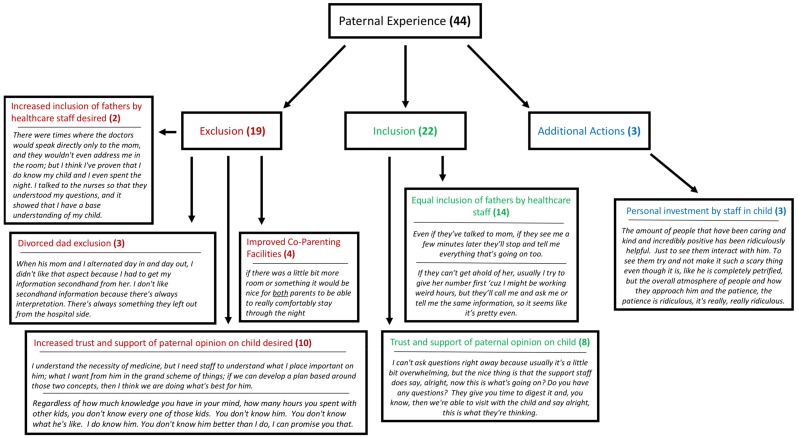
Flow chart depicting the paternal experience within the healthcare setting categorized into exclusion, inclusion, and additional actions. The bolded number represents the amount of extracted codes identified to match the respective theme, and the italics below each theme are specific quotations taken from paternal interviews.

**Figure 2:**
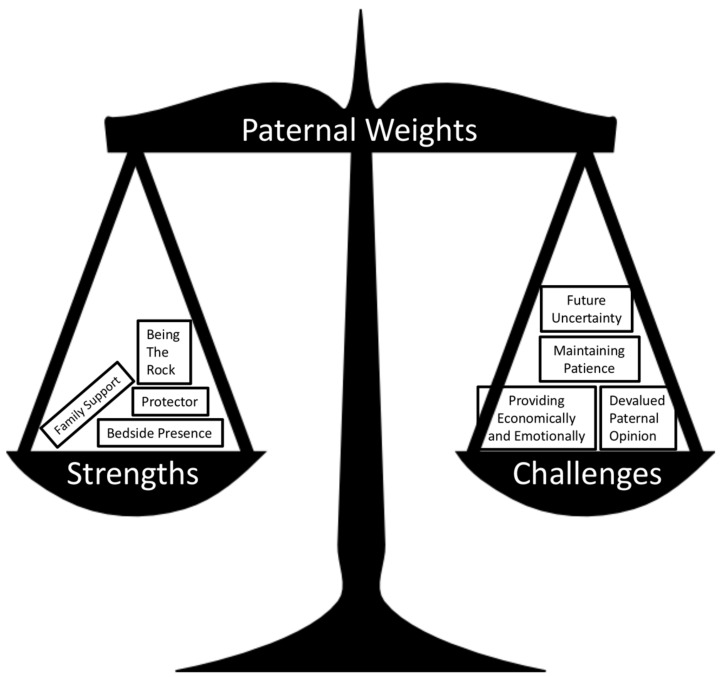
Symbolic representation of the conflicting weights experienced by fathers in their care for their child. Each box is a specific theme extracted from paternal interviews.

The psychosocial effects of a pediatric brain tumor diagnosis and treatment course impact the entire family unit, including the child’s parental figures [1,2,3]. Parental caregiver distress is notably higher in pediatric brain tumor diagnoses than for parents of children with other forms of cancer [4]. The multiple psychosocial impacts of childhood brain cancer are noted to have lasting and late effects, not only for survivors of childhood brain tumors, but also for their parents [1,5,6,7]. Parents of survivors of childhood brain tumors describe, “an intensification of the parenting role, a different way of being, and the need for additional help” [8]. The Standards of Psychosocial Care for Parents of Children with Cancer call for personalized, attentive care models for the parents of children with cancer as a component of quality pediatric oncology care [9].

Parents of brain tumor patients experience distress in excess of other childhood cancer parents, as noted by the issues unique to neuro-oncology diagnoses as compared to other childhood cancers: higher mortality rates; clustering of symptom burden such as fatigue, headache, pain, nausea, weakness, changes in ambulation and strength; faster progression; lasting changes in the child’s cognition and development; and impact to the child’s personality. Thus, it important to study these parents separately to assess their unique care needs. The experience of mothers has been described and delineated between mothers of children living with brain tumors [10] and bereaved mothers of children with neuro-oncologic diagnoses [11]. Fathers have been notably under-represented in studies of parents of children with brain tumors [1]. Investigations into parental caregiver adaptation and support interventions have consistently under-explored paternal experience [9], and yet, paternal experience is recognized as unique in meaningful ways from maternal experience. Use of latent class analysis to categorize beliefs representing a parent’s perception of what they should do to be a good parent for a seriously ill child revealed a gender-based grouping of fathers to include a paternal emphasis on information-gathering about the child’s health and making informed care decisions [12]. An international, two-year longitudinal study of 88 parents of children with brain tumors revealed the uniqueness of coping strategies and support needs specific for fathers, revealing paternal focus on work and employment as a form of “coping”, and fathers relying on pre-existing support networks such as brothers, colleagues, and childhood friends [13]. A systematic review across studies on pediatric neuro-oncology cohorts found that fathers reported healthcare providers tend to address mothers as primary caregivers, which leads fathers to feeling less informed and less included [14]. This qualitative case series offers insight into paternal-specific roles, responsibilities, and support needs for fathers of a pediatric neuro-oncology cohort who have received a new palliative care consultation.

## 2. Materials and Methods

The Institutional Review Board (IRB) approved the Paternal Advice, Guidance, Education, and Support (PAGES) protocol. The study reporting followed strict Consolidated Criteria for Reporting Qualitative Research (COREQ) guidelines (Supplementary Materials 1). Informed consent was obtained from all individual participants included in the study.

### 2.1. Sample Description

This qualitative study was a pilot approach. Four fathers of children undergoing treatment for a neurological malignancy participated in a one-on-one, one-timepoint, voice-recorded, in-person interview at a free-standing children’s hospital in the Midwestern United States. Individual interviews were completed from December 2018 to February 2019. Eligibility criteria included English-speaking fathers of children undergoing treatment for a neuro-oncological diagnosis with new palliative care consultations. Sampling occurred in a sequential convenience sampling manner based on palliative care team consultations for pediatric neuro-oncology patients. Fathers were excluded from the study if the father declined to participate at any time of the recruitment or interview process.

### 2.2. Study Procedures

Prior to beginning the interview, participants were provided a full study description, and verbal and written consent for participation was obtained. All interviews were audio-recorded and conducted in a private hospital inpatient or outpatient room by a trained research associate, who had no previous connection to the child or their family. Three paternal interviews occurred in the inpatient hospital setting, while one father was interviewed while their child received outpatient treatment in a private infusion room. The interviews were semistructured using an interview guide (Supplementary Materials 2), as has been suggested as the standard approach for cognitive interviewing targeting conceptual definitions [15]. Interviews lasted 20 min on average, and afterwards a “content check” was completed by the study team for each participant to provoke processing of the interview experience by the interviewees [16]. The “content check” consisted of the study team summarizing the information the interviewee had shared and confirming with the interviewee that the summary was correct. Audio recordings were transcribed by trained medical transcriptionists, and each transcription was verified for its accuracy by at least one researcher prior to analysis. The fathers were specifically asked to define “what does it mean to be a good dad for your child?” and to then describe their paternal sense of roles, responsibilities, strengths, challenges, information needs, and medical team support preferences that would enable them to reach that “good dad” definition.

Immediately following the interview, participants completed a two-page, multiple choice survey with questions regarding demographics and preferences for formatting and content of potential informational resources. The participating fathers ranked their overall information needs regarding their child’s care when presented with a 1–5 Likert scale (with one representing “low knowledge need” and five representing “high knowledge need”). The fathers were then asked to rank their information preferences on a subtopic of information needs, with the medical condition to include prognostic or treatment information, emotional support, and grief support.

### 2.3. Data Analysis

Interview data was analyzed using semantic content analysis [16,17]. One member of the study team identified key phrases from interview responses to each question and applied codes to capture their meaning. These codes were then grouped and classified in order to form a code dictionary, which consisted of the definition and a quoted example of the code from the interviews. Coded phrases that were incidental responses to previous questions were moved to the appropriate question if agreed upon by at least two researchers. A second and third researcher then utilized this code dictionary to independently review interview data so as to validate coding for each phrasing. Upon confirmation of coding, themes were established by grouping codes with similar meaning and function. These themes were then provided and corroborated using quotes and codes of the original data and its analysis.

The written survey was revised from a prior published survey inquiry into grandparent knowledge and support needs [18]. Written survey results were descriptive and univariate. The mean, median, and average was calculated and provided when appropriate; otherwise, counts for categorical variable responses were obtained.

## 3. Results

This exploratory qualitative interview study included a case series of four Caucasian fathers to two female and two male patients with brain tumors receiving palliative care consultations. Five eligible participants were approached during the study timeframe based on convenience sampling, with *n* = 4 enrollment (80% participation rate). One father declined participation in the interview because of fatigue at the time of approach. Complete demographic listings of participating fathers and their child can be found in Table 1.

### 3.1. Paternal Knowledge Content and Format Preferences

Fathers ranked their overall information needs regarding their child’s care as 4.5/5 on the 1–5 Likert scale. Participants unanimously ranked topics regarding their child’s medical condition, such as prognostic and treatment information, as highest informational priorities (mean score 5/5). The fathers ranked information regarding their own emotional support needs (mean score 2.3/5) and grief or bereavement support (mean score 2.7/5) as the lowest knowledge-seeking prioritizations. Each interviewee preferred conversation via support group or private discussion as the mechanism of receiving information. Three of the four fathers requested a written booklet for information sharing partnered with in-person conversation.

### 3.2. Self-Definition of Paternal Role in Caring for a Child with a Neuro-Oncological Diagnosis

When asked to define the role of a father in a child’s care, 19 phrases matching 27 codes were identified that aligned with 6 themes. Paternal role themes were depicted as being a team parent, an adaptable father, a supportive father, a provider, a present father, and a protective father. “Being a present father” was a theme identified by three different interviewees, ranking as the most prevalent theme. “Being a supportive father” was ranked as the most pressing theme. One father stated the importance of being supportive as: “I have to keep him to where he does not lose faith in me, he does not lose faith in this hospital, and he does not lose faith in why we are doing something, because then he just gives up. If he gives up, it is over.” Paternal narrative emphasized the self-perceived role of supporting the child and the family through the child’s treatment, even if the father’s employment responsibilities prevented the father from being physically present.

Fathers were asked to define what it would mean to be a “good dad” for their child at this time in their life. Four major themes were identified containing a total of 27 codes (Table 2). One participant was a single father who referenced the need to remain supportive of the child’s relationship with their mother when asked how he defined a “good dad”. Fathers utilized language of selflessness and both provider and protector when depicting their definition of good fathering for an ill child. The protector theme included protection from physical pain or symptom burden. The provider theme included provision of playtime, distraction, humor, and more tangible provisions, such as health insurance and housing security.

### 3.3. Hospital Actions of Paternal Inclusion and Exclusion

Participants were asked four separate questions that aimed to encompass their overall experience as fathers within the healthcare system, particularly in terms of their inclusion or exclusion from their child’s care. A total of 44 codes were identified among the interview phrases. The codes were categorized as feelings and actions of inclusion, feelings and actions of exclusion, or additional hospital supportive actions (Figure 1). Many codes classified within the inclusion or exclusion categories were dichotomous to one another, demonstrating the heterogeneity of individual paternal inclusion experiences, despite the common desire for support between all fathers. Fathers’ inclusion or exclusion was not unilateral, as 3 of the 4 fathers recalled situations of inclusion, and 3 of the 4 fathers also recounted situations of exclusion.

### 3.4. Unique Paternal Strengths and Challenges

Fathers were asked about stresses, challenges, and individual strengths that are unique to them. Finding common strengths and challenges among the four participants is challenging due to the heterogeneity of each father’s experience. Patterns of intrapersonal stress, turmoil, and conflict were related to the competing demands, everyday stresses of caregiving, and the pressure of meeting family and societal expectations and norms. In response to intrapersonal stress, the interviewed fathers depicted creativity, strong resolve, patience, anxiety, and fear of the future. The participants repeatedly noted that their vulnerabilities and strengths must be balanced in order to continue serving as “the rock” (Figure 2) for their family unit.

### 3.5. Personal Growth

Eleven statements were made by fathers reflecting on their personal growth as a result of their child’s diagnosis: five statements were of an intrapersonal nature and six statements related to interpersonal growth. Themes of intrapersonal growth were appreciating fatherhood (2 statements), aspiring into fatherhood (1 statement), and prioritizing fatherhood (2 statements). Interpersonal statements were in regard to growing in patience (2 statements) and developing resiliency (4 statements).

Among intrapersonal growth, participants grew into better fathers for their child and family. Fathers referenced having a greater appreciation for each day with their child. As one stated, “I think that especially with what is happening now, understanding how short time is, how short time can become, and just saying we cannot predict what is going to happen. Let us do something. Let us push forward and let us give them (the kids) a memory regardless of how it comes out. It is their memory. Let us just try and make one.” Additionally, fathers described prioritizing fatherhood more than other relationships in their lives (such as other male friendships) as a result of their child’s condition.

The interpersonal growth described was in reference to the fathers’ improved patience or developed resiliency. One stated, “I am not one to sit still very long”, and proceeded to state that he had grown in his patience and in his ability to remain attentively present to his child due to the many days he was spending in the hospital with his child. Two fathers referenced their improved resiliency as a result of their child’s diagnosis. They described being “pushed to the limits” and “being knocked down” by the child’s brain tumor diagnosis and then having to “overcome” and “get back up” to pursue treatment. A consistent interview theme was recognition of the heavy emotional toll the situation was taking on the father, partnered with a willing commitment to persevere for the child. Although fathers verbalized a message of patience and resiliency, there was also an unspoken undercurrent of sacrifice and selflessness in the paternal narratives.

### 3.6. Advice for Other Fathers

Interviewees were asked to provide advice that they would offer to other fathers of children diagnosed with brain tumors on “how to be a good dad”. The advice included supporting the child and overcoming adversity. Fathers advised supporting the child by ensuring adequate insurance coverage, being physically present for their child, or connecting with the child emotionally and playfully. One father’s advice was simply to, “love, just love (them) as much as you can.” Two fathers specifically shared advice on facing adversity in their situation; finding the positives, embracing the present and future challenges, and seeking an emotional outlet. One stated, “Everybody has challenges. You have just got to, you know, be a man, step up, and take care of your responsibilities.” The fathers advised to not be ashamed to show emotions. A father phrased it, “You know, men are supposed to be the strong ones. We are supposed to not show emotion. We are supposed to be that standing rock.” Another stated, “You can bury down as long as you want. You can push it (emotion) away and try not think about it or just ignore it. But, eventually, it is going to come back to the surface. Deal with it.” The paternal narratives recognized the difficulty in expressing feelings as a father, however, also realized the importance of expressing those emotions in such a poignant time.

## 4. Discussion

The concept of family resiliency requires inclusive biopsychosocial frameworks that elicit not just maternal but also paternal voices to improve overall child and family health outcomes. As palliative care teams emphasize family coping and decision-making by exploring patient and family values [19], this qualitative case series explored the self-defined “good dad” role to explore paternal responsibilities and needs. This case series revealed a tension between the paternal need to be strong and the raw emotional experiences of fathers of children with brain tumors. In a phenomenological inductive study of family experience with pediatric cancer diagnoses, fathers reported “feeling powerless” [20] despite feeling a need to remain strong. In a focus group methodology study for fathers of children with cancer, many described their “attempt to remain in control despite feeling vulnerable” [21]. The results from this case series study similarly depict fathers struggling with emotion, and yet emotional support needs were self-identified as the lowest prioritized knowledge need on the quantitative survey, seemingly because the fathers in this case series were prioritizing the direct support needs of their children and other family members, such as their co-parent.

The interdisciplinary neuro-oncology team should specifically consider the distress the child’s diagnosis brings to a child’s father. Fathers (*n* = 72) of children with cancer reported significant increase in traumatic stress symptom and decrease in social support between time of diagnosis to check-in four months later [22]. Forty percent of fathers (*n* = 27) of adolescents with cancer were noted to have state anxiety scores more than one standard deviation above the population mean score, with 38% of fathers reporting mild to moderate depression [23]. Fathers do have emotional and support needs, although there is a struggle to seek or access support.

The ideas of how a father defines his paternal role and how a father defines “being a good dad” notably result in overlap responses, as both are a form of paternal identity. A father role is notably a more sterile question, as the question relates to biological duty, whereas the “good dad” concept embodies the relational component of interaction with the child and the virtues or values upheld in this personal role.

Traditionally, a father’s process of coping with a child’s complex, chronic, life-limiting illness has been reported to focus on solutions and pragmatic, action-oriented strategies, such as information gathering or financially providing [24]. Without understanding of the potentially unique approach of fathers as compared to mothers, paternal coping could be misinterpreted as avoidance or denial as compared to the more emotive or affective maternal response [25]. In a study of fathers of children with cancer, the *n* = 20 fathers of children with cancer were noted to spend more hours at work and more hours caring for other children than the *n* = 20 control group fathers [26]. Whether this is an avoidant coping strategy or a goal of serving as financial provider is difficult to extrapolate from quantitative data. Another possibility is that the parents have divided their roles, with the mothers focusing on the sick child in the hospital while the father focuses on continuing to maintain family income and care for the other children, strategically enabling the mother to focus one-on-one attention to the child with a brain tumor diagnosis while the father cares for additional family needs. In a grounded theory qualitative study of *n* = 16 fathers of children being actively treated for cancer, they defined the role as “providing family support, providing resources, as well as maintaining family stabilization.” [27]. This study shared paternal sense of responsibility for economic security and “remaining strong” for their spouse. In a systematic review of research studies on fathers of children diagnosed with cancer, employment concerns and the frequency of hospital admissions were noted as significant stresses for fathers of children with cancer across multiple studies, presumably as these impacted the paternal provider/protector role [14].

Paternal knowledge need was notably high in this study’s written survey findings, particularly regarding the child’s diagnosis, prognosis, and treatment plan. Access to medical information and clear communication has been emphasized in qualitative reports from fathers of hospitalized children with brain tumors [28]. Neuro-oncology teams are prudent to offer to share information with fathers who may not be able to attend physical appointments, both to lift medical message interpretation from the co-parent and to gesture proactive inclusion of both parents. Provision of written information or workbook formats of information sharing may be particularly supportive for co-parent communication.

The findings from this small case series run parallel to the findings of a qualitative interview of maternal perception of paternal coping and paternal self-perception of coping in their parenting of children with leukemia. The leukemia cohort qualitative study revealed that: several fathers found it difficult to share their emotions and specifically named strategies to avoid public emotion; fathers described responding to the stress of their child’s cancer by withdrawing from the situation or environment; fathers described using blocking or avoidance strategies when dealing with tension; fathers felt the need to maintain a positive attitude to set the tone for the rest of the family [29].

Limitations of this case series work include a small study sampling of a homogenous nature. Because of the small sample size and lack of racial and ethnic diversity, the themes should not be assumed to represent the spectrum of experiences of all fathers. The small sample prevents summary assessment of universal experiences versus experiences that are less common. The strengths of the study include rigorous application of qualitative methodology and inclusion of paternal perspective.

Future studies should include longitudinal exploration across the duration of diagnosis, treatment, and ultimately cure or bereavement to better understand how role definitions and role priorities change or evolve over time. Future studies should include additional family compositions, such as biological fathers and step-fathers. Future studies should consider dyad-questions to understand how maternal and paternal figures identify their sense of “virtuous care” within their family role.

## 5. Conclusions

Healthy family function and access to personalized family support are noted to be protective factors in outcomes for pediatric brain tumor survivors [30,31]. This qualitative case series provided insight into the self-perceived paternal role, responsibilities, personal growth, and information needs, and support preferences of fathers of children with brain tumors. Fathers of children diagnosed with a brain tumor may be heavily impacted by their child’s diagnosis and treatment plan, despite using self-depicting language such as “family foundation” and “the rock” to describe their roles. Many fathers carry multiple demands and often take on new roles in the family during their child’s care. Fathers of pediatric neuro-oncology patients may be struggling to balance the demands of financial responsibility, hands-on caregiving, and supporting additional family members’ emotional and practical, day-to-day needs while remaining present to medical teams [32]. Healthy family functioning requires eliciting family roles and goals, and mobilizing the necessary resources to facilitate caring and inclusion of parental caregivers for overall family strengthening.

## Figures and Tables

**Table 1:** Participant demographics.

Characteristics		*n* = ____ (%)
Child’s Gender	Male	2 (20)
	Female	2 (20)
Child’s Age	Mean/Range (years)	9.25/5–14
Child’s Time Since Diagnosis	Mean/Range (months)	47.8/3–168
Child’s Location at Time of Interview	Hospital	3 (75)
	Outpatient	1 (25)
Father’s Ethnicity	Caucasian	4 (100)
Father’s Home Locale	Rural	1 (25)
	Urban	3 (75)
Distance Home to Hospital	Mean/Range (miles)	31.5/10–90
Does the Child Reside Within the Home?	Yes	4 (100)
Number of Other Children Living in the Home	Mean/Range (children)	3.33/0–5
Parental Marital Status	Married	3 (75)
	Separated	1 (25)
Parent Highest Level of Formal Education	Some College	2 (50)
	College Completion	2 (50)
Parent Employment Type	Manual Labor	2 (50)
	Office Work	2 (50)
Primary Caregiver of Child	Both	2 (50)
	Father	1 (25)
	Mother	1 (25)

**Table 2:** Paternal definition of “being a good dad to my child”.

Themes (*n* = 4)	Definition	Codes, *n* = __/ 27 (%) Participants, *n* = __/ 4 (%)	Exemplary Quotes
Teacher	A “good dad” is a willing teacher for his child. He teaches them values and lessons, and he provides the opportunity for them to achieve their life aspirations.	4 (14.8%), 2 (50.0%)	“If you want to be an astronaut on Mars, go for it. It is going to take work. That is my job as a father to give them those, give them access to those avenues.” “Try to teach them as much as I can.”
Fulfiller of Life	A “good dad” provides a wide array of life experiences for their child. He helps them achieve their goals, and he helps them enjoy life through fun adventures and happy moments.	11 (40.7%), 3 (75.0%)	“Giving them 100% access to the things that make them, allow them to figure out who they are.” “You know, like take them camping, fishing, and let them see everything.” “Making sure she gets as many experiences as possible and having a full life.” “(Providing) life, like trying different foods.”
Puts Child First	A “good dad” is unselfish. He puts his needs behind the needs of his child and his family, and he is present and involved with them for their entire life.	10 (37.0%), 2 (50.0%)	“Somebody who puts the needs of the family above their needs and their wants.” “I just think that it just takes time and you have to be involved and you have to be willing to, you know, takes days off of work. You have to be willing to put what you can from your career off to the side.”
Rises to the Occasion	A “good dad” rises to the occasion of supporting their child by doing everything they may need to and by being strong, by “being a man.”	2 (7.4%), 2 (50.0%)	“(Help them) with everything.” “That is the role of being a man.”

## Supplementary Materials

The following are available online at https://www.mdpi.com/2227-9067/6/10/113/s1. Supplementary Materials 1: Consolidated Criteria for Reporting Qualitative Research (COREQ) guidelines; Supplementary Materials 2: Qualitative Interview Guide.
