# Supplementary material for: The Many Roles of the Rock: A Qualitative Inquiry into the Roles and Responsibilities of Fathers of Children with Brain Tumors
Source: Children (Basel). 2019 Oct 11;6(10):113. doi: 10.3390/children6100113 (PMC6826713; doi:10.3390/children6100113)
Supplement: Supplementary file 1 [file children-06-00113-s001.zip › Supplementary Material/Supplemental Material 1_COREQ_Qual Guidelines.pdf]

Supplemental Material 1.

Consolidated criteria for reporting qualitative studies (COREQ): 32-item checklist

**Domain 1. Research team and reflexivity**	
**Personal characteristics**	
1. Interviewer/facilitator	JR conducted the interviews<br>MW visited with interviewees after the interviews to confirm content
2. Credentials	The study team consisted of doctoral-level investigators from the field of pediatric palliative care, chaplaincy, and masters-level investigators from the field of social work and research
3. Occupation	Members of the study team included physician, chaplain, social work, and research occupational backgrounds
4. Gender	The research team consisted of a balance of gender representation with a heavier proportion of male members due to paternal nature of study topic
5. Experience and training of interviewer	JR has completed cognitive interview mentorship from MW<br>MW has completed >30 hours of formal interview training from sociology/anthropology investigators, has completed qualitative software coursework with tutelage from an Atlas.ti software designer, and completed graduate-level qualitative methodology coursework prior to study design and interview implementation<br>To experience paternal perspective, study team members met with colleagues with each colleague assigned one transcript and shared their reflections of that one paternal perspective/experience in group format to “be the voice” of that interviewed dad prior to research analysis
**Relationship with participants**	
6. Relationship established	The study was mentioned to the case family by their palliative care physician at least two days prior to interviews with an in-person introduction to interviewer occurring the day of the interview
7. Participant knowledge of interviewer	The participants were made aware that the interviewer is interested in paternal role, responsibility, and support need constructs and that the study team was interested in learning about the family’s experiences to gain insight about how best to support paternal roles
8. Interviewer characteristics	The interviewer utilizes positive affirmation during interviews (nodding and sharing verbal agreement) with follow-up questions (while avoiding leading) and tolerates extended silence during interviews to foster open, engaged conversations
**Domain 2. Study design**	
**Theoretical framework**	
9. Methodological orientation and theory	Case study methodology underpinned the study; semantic content analyses was applied for data review<br>Study team members engaged in a reflective meeting prior to research meetings at which time study team member reflected on their own definitions and perceptions of fatherhood from their personal and clinical experiences
**Participant selection**	
10. Sampling	The participants were selected based on consecutive neuro-oncology cases and convenience sampling
11. Method of approach	Participants were approached face-to-face
12. Sample size	n=4
13. Non-participation	Four of five invited participants elected to participate after an engaged process of informed consent; one of five invited participants declined due to fatigue

**Setting**	
14. Setting of data collection	Data was collected in private hospital or outpatient clinic rooms
15. Presence of non-participants	Participant and the researcher were present in the interview room
16. Description of sample	Demographics and data are deliberately omitted from responses to protect the privacy/confidentiality of participants
**Data collection**	
17. Interview guide	The questions were reviewed by one psychologist expert from a different institution and one family-theorist from a different institution
18. Repeat interviews	Repeat interviews were not carried out
19. Recording	Interviews were voice recorded
20. Field notes	Field notes were not made during the interviews, but, the interviewer engaged in memoing immediately after the interviews to reflect upon interview setting/content
21. Duration	The interviews lasted an average of 20 minutes
22. Data saturation	Saturation was not an a priori goal; any and all raised themes were included as relevant
23. Transcripts returned	Content was reviewed by participants with MW as an opportunity for debriefing from the interview experience
Domain 3. Analysis and findings	
24. Number of data coders	Two data coders coded the data
25. Description of coding tree	Coding tree included mapping of themes and relationships between themes by the study team in in-person meetings
26. Derivation of themes	Themes were identified from the data as social constructs
27. Software	Excel was used to manage the data
28. Participant checking	Participants provided feedback on findings through content checking after the interviews were complete
**Reporting**	
29. Quotations presented	Participant quotations were presented to illustrate themes; to protect participant privacy, quotation was not identified by participant name.
30. Data and findings consistent	The data presented was consistent with evidence base gathered through literature search completed prior to interview initiation in prior Good Parent qualitative manuscripts
31. Clarity of major themes	Major themes were clearly presented in the form of text summary and a table
32. Clarity of minor themes	Minor themes were described by text summary, list format, and a table in the manuscript
