# Supplementary material for: The Many Roles of the Rock: A Qualitative Inquiry into the Roles and Responsibilities of Fathers of Children with Brain Tumors
Source: Children (Basel). 2019 Oct 11;6(10):113. doi: 10.3390/children6100113 (PMC6826713; doi:10.3390/children6100113)
Supplement: Supplementary file 1 [file children-06-00113-s001.zip › Supplementary Material/Supplemental Material 2_Interview Guide.pdf]

Supplemental Material 2. Qualitative Interview Guide

1. As a dad, how do you define your role in the care of your child with a brain tumor?
2. Please share with me your definition of being a good dad to your child with a neuro-oncology diagnosis at this point in your child's life?
3. What sort of personal growth have you experienced in your fatherly role from the time of your child's brain tumor diagnosis to now?
4. Please share about your overall experiences with the healthcare system as a dad – particularly in terms of your inclusion?
5. What have health care team members done or said that helped you to feel most included as a dad?
6. What behaviors that have left you feeling excluded in your child's care?
7. Please describe for me the actions from staff that would help you in your efforts to be a good dad to your child now (supportive actions from staff).
8. What advice would you give to other fathers who have a child with a brain tumor?
